# PACC1 could serve as a prognostic biomarker for patients with hepatocellular carcinoma

**DOI:** 10.1097/JS9.0000000000004632

**Published:** 2026-01-07

**Authors:** Zibin Wang, Jiajia Bian, Jiankun Zhang, Liping Zhang

**Affiliations:** a Department of General Surgery, Hebei Petro China Central Hospital, Langfang, China; b Department of Anesthesiology, Hebei Petro China Central Hospital, Langfang, China; c Department of Urology, Hebei Petro China Central Hospital, Langfang, China

Liver cancer is the sixth most common malignancy worldwide and ranks second in cancer-related mortality^[1]^. Primary liver cancer includes three major pathological subtypes: hepatocellular carcinoma (HCC), intrahepatic cholangiocarcinoma (iCCA), and the less frequent combined hepatocellular-cholangiocarcinoma (cHCC-CCA). According to the GLOBOCAN 2022 statistics, HCC accounts for approximately 75%–85% of cases, whereas iCCA represents 10%–15%^[2]^. Owing to its asymptomatic early stages, rapid progression, and low rate of early detection, most LIHC patients are diagnosed at advanced stages, missing the optimal window for effective treatment. This highlights the critical need to identify novel biomarkers that could serve both prognostic and therapeutic purposes. Proton-Activated Chloride Channel 1 (*PACC1*, also known as TMEM206) is a member of the transmembrane (TMEM) protein family^[3]^, several of which have been implicated in tumor initiation, progression, and metastasis^[4]^. Notably, *PACC1* has been shown to contribute to key oncogenic processes in colorectal cancer and is regulated by p53 in a p21-dependent manner^[5]^. This manuscript is conducted in accordance with the TITAN Guidelines 2025^[6]^.

To elucidate the potential function of *PACC1* in liver cancer, we analyzed transcriptomic profiles and matched clinical information from The Cancer Genome Atlas (TCGA-LIHC). We assessed *PACC1* expression patterns, explored its prognostic significance, and performed Gene Ontology (GO) and Kyoto Encyclopedia of Genes and Genomes (KEGG) functional enrichment analyses. Furthermore, we investigated its association with the tumor immune microenvironment using multiple independent computational algorithms.

Table 1 summarizes the baseline clinical characteristics of LIHC patients. Pan-cancer analysis revealed heterogeneous *PACC1* expression, with a pronounced upregulation in LIHC tumor tissues relative to normal samples (Fig. 1A-E). High *PACC1* expression was significantly associated with advanced histologic stage and T stage (Fig. 1F-G), while statistically significant, the T-stage association is modest and should be interpreted cautiously. Kaplan–Meier survival analysis demonstrated that patients with elevated *PACC1* levels had significantly poorer overall survival compared to those with low expression (Fig. 1H). ROC curve analysis showed strong diagnostic potential, with an AUC of 0.963 (Fig. 1I). Gene Ontology (GO) and Kyoto Encyclopedia of Genes and Genomes (KEGG) analyses indicate that *PACC1* may be involved in LIHC development through various biological processes and pathways, such as detoxification of copper ion, stress response to copper ion, axonogenesis, mineral absorption, neuroactive ligand–receptor interaction, metabolism of xenobiotics by cytochrome P450, retinol metabolism, ECM–receptor interaction, and IL-17 signaling pathway (Fig. 1J-K). Immune infiltration analysis using five independent algorithms (CIBERSORT, EPIC, MCPcounter, quanTIseq, and TIMER) consistently linked *PACC1* expression with altered immune cell landscapes (Fig. 1L-P). Single-cell RNA-seq analysis further revealed that *PACC1* is predominantly expressed in natural killer (NK) cells and CD8^+^T cells in dataset GSE179795, whereas its expression was not detected in dataset GSE125449, suggesting that *PACC1* expression may change following PD-L1-targeted therapy (Fig. 1Q-T).

**Figure 1.**
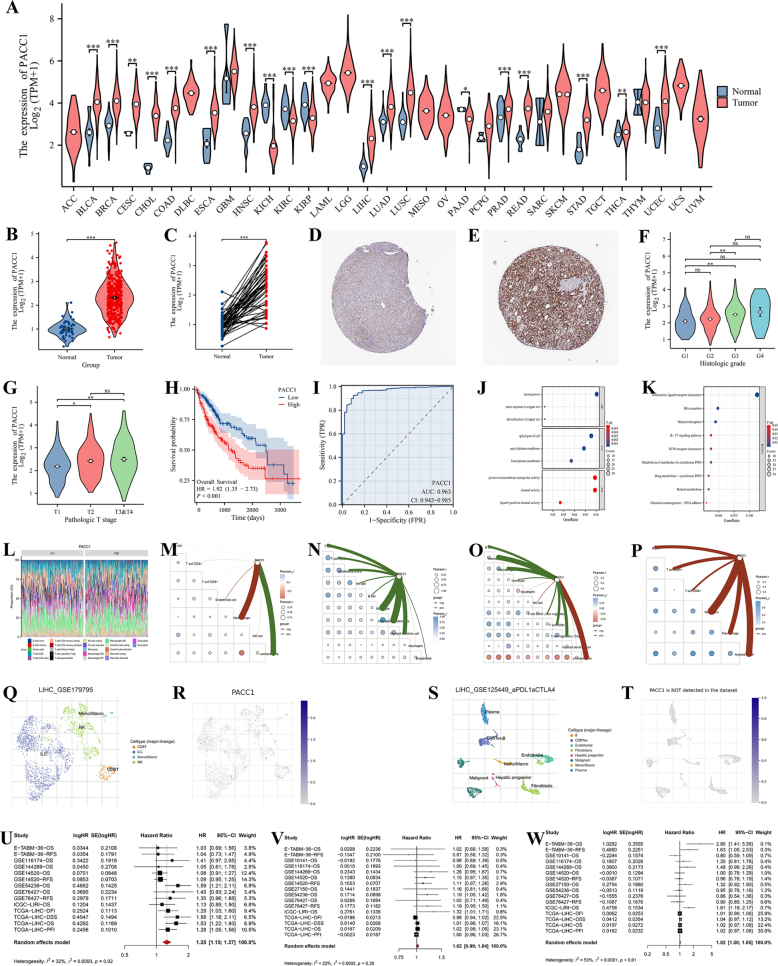
(A) Pan-cancer expression of PACC1 compared with normal tissues based on TCGA data. (B–C) PACC1 expression in unpaired (B) and paired (C) LIHC samples. (D–E) Immunohistochemical staining from the HPA database showing PACC1 in normal liver tissue (D) and LIHC tissue (E). (F–G) Correlations between PACC1 expression and histologic stage and T stage. (H) Kaplan–Meier analysis of overall survival in LIHC patients stratified by PACC1 expression. (I) ROC curve analysis evaluating the diagnostic performance of PACC1. (J–K) GO and KEGG enrichment analyses of differentially expressed genes associated with PACC1 in LIHC. (L–T) Assessment of tumor immune microenvironment and immune cell infiltration linked to PACC1 expression. Statistical signifcance is indicated as *P < 0.05, **P < 0.01, ***P < 0.001.

**Table 1:** Association of PACC1 expression with clinicopathological features in TCGA-LIHC.

Characteristics	Low expression of PACC1	High expression of PACC1	*P*-value
*n*	186	188	
Pathologic T stage, *n* (%)			0.038
T1	103 (27.8)	80 (21.6)	
T2	42 (11.3)	53 (14.3)	
T3&T4	39 (10.5)	54 (14.6)	
Pathologic N stage, *n* (%)			0.681
N0	122 (47.3)	132 (51.2)	
N1	1 (0.4)	3 (1.2)	
Pathologic M stage, *n* (%)			1.000
M0	126 (46.3)	142 (52.2)	
M1	2 (0.7)	2 (0.7)	
Gender, *n* (%)			0.356
Female	56 (15)	65 (17.4)	
Male	130 (34.8)	123 (32.9)	
Albumin(g/dL), *n* (%)			0.192
<3.5	42 (14)	27 (9)	
≥3.5	120 (40)	111 (37)	
Histologic grade, *n* (%)			< 0.001
G1	37 (10)	18 (4.9)	
G2	97 (26.3)	81 (22)	
G3	47 (12.7)	77 (20.9)	
G4	4 (1.1)	8 (2.2)	
Residual tumor, *n* (%)			0.038
R0	173 (50.1)	154 (44.6)	
R1&R2	5 (1.4)	13 (3.8)	

Figure 1U-W presents a forest plot of the meta-analysis integrating univariate Cox proportional hazards results from TCGA-LIHC and several independent external datasets. The combined results confirm that high *PACC1* expression is significantly associated with poorer survival outcomes in liver cancer patients. Notably, its prognostic performance appears to exceed that of widely used biomarkers such as alpha-fetoprotein (AFP) and glypican-3 (GPC3).

In conclusion, our study suggests that *PACC1* may function as a promising biomarker for both diagnosis and prognosis in LIHC. *PACC1*, as a chloride channel, may influence cellular stress responses, ion homeostasis, and tumor–immune interactions, which could plausibly link copper stress, xenobiotic metabolism, and immune pathways. These findings warrant further investigation into the potential of *PACC1* as a therapeutic target in LIHC. As this study is based solely on bioinformatic analyses, further *in vivo* and *in vitro* investigations are warranted to validate the mechanistic functions of *PACC1* and assess its potential as a therapeutic target in liver cancer.

## Data Availability

All data are obtained in the article.

## References

[1] Valery PC Laversanne M Clark PJ Petrick JL McGlynn KA Bray F. Projections of primary liver cancer to 2030 in 30 countries worldwide. Hepatology (Baltimore, MD) 2018;67:600–11. 10.1002/hep.29498 PMC5832532 28859220

[2] Bray F Laversanne M Sung H. Global cancer statistics 2022: GLOBOCAN estimates of incidence and mortality worldwide for 36 cancers in 185 countries. Ca: A Cancer J Clin 2024;74:229–63. 10.3322/caac.21834 38572751

[3] Chen DL Cai JH. and Wang CCN. Identification of key prognostic genes of triple negative breast cancer by LASSO-based machine learning and bioinformatics analysis. Genes 2022;13: 902. 35627287 10.3390/genes13050902 PMC9140789

[4] Marx S Dal Maso T Chen JW. Transmembrane (TMEM) protein family members: poorly characterized even if essential for the metastatic process. Semin Cancer Biol 2020;60:96–106. 31454669 10.1016/j.semcancer.2019.08.018

[5] Melek K Hauert B Kappel S. TMEM206 contributes to cancer hallmark functions in colorectal cancer cells and is regulated by p53 in a p21-dependent manner. Cells 2024;13:1825. 39594575 10.3390/cells13221825 PMC11593115

[6] Riaz AA Ginimol M Rasha R. Transparency in the Reporting of Artificial Intelligence – the TITAN Guideline. Prem J Sci 2025;10:100082.

